# Comprehensive evaluation of agronomic, processing, nutritional and functional properties of 22 main-cultivated adzuki bean varieties in China and identification of elite varieties for different applications

**DOI:** 10.3389/fpls.2026.1947671

**Published:** 2026-09-04

**Authors:** Lei Zhang, Zhaojin Shi, Congpei Yin, Fan Jia, Yuancong Li, Hongmei Zhou, Qiang Cui, Jiaxing Li, Dongxiao Li, Yuechen Zhang

**Affiliations:** 1 College of Life Sciences, Zaozhuang University, Zaozhuang, China; 2 State Key Laboratory of North China Crop Improvement and Regulation/Key Laboratory of Crop Growth Regulation of Hebei Province, College of Agronomy, Agricultural University of Hebei, Baoding, Hebei, China; 3 Baoding Academy of Agricultural Sciences, Baoding, China; 4 Hezhuang Primary School, Tanshan, China

**Keywords:** adzuki bean, genetic diversity, principal component analysis, quality evaluation, special-purpose variety

## Abstract

Adzuki bean (*Vigna angularis*) is an important medicinal and edible legume with high nutritional and functional value. However, a systematic multi-dimensional quality evaluation system for mainstream varieties remains insufficient, restricting targeted breeding and industrial diversified utilization of adzuki bean. This study comprehensively assessed the agronomic, nutritional, processing and functional traits of 22 dominant Chinese adzuki bean varieties to screen promising candidate germplasms for specific applications. Multiple indices covering agronomic traits, mineral nutrition, seed color, processing quality and antioxidant capacity were determined, and 18 core indices were analyzed via principal component analysis (PCA). Results revealed abundant phenotypic variation across all traits, with pods per plant, isoflavone monomers and soluble sugar showing the most pronounced phenotypic variation. PCA ranked Baihong 2, Jihong 8 and Jihong 13 as the top three varieties under the tested conditions, and identified special-purpose cultivars including high-yield Jihong 9218, isoflavone-rich Baohong 8824 and antioxidant-rich Kenjianhong. This study established a multidimensional quality evaluation framework, providing scientific reference for promising cultivar screening, targeted breeding and sustainable industrial development of adzuki bean.

## Introduction

Adzuki bean (*Vigna angularis*) is a traditional edible legume native to China with a cultivation history exceeding 2000 years across East Asia (Xu et al., 2008). As a typical medicinal and edible crop, its seeds are abundant in high-quality protein, complex carbohydrates, mineral elements and bioactive phytochemicals, possessing antioxidant, anti-inflammatory, hypoglycemic and gut microbiota-regulating physiological functions (Luo et al., 2016). Growing global demand for natural healthy foods has driven the continuous expansion of the adzuki bean processing market, raising higher comprehensive quality requirements for commercial cultivars. Conventional adzuki bean breeding programs have long prioritized grain yield, while insufficient attention has been paid to processing suitability, nutritional composition and functional characteristics. Such one-sided breeding orientation has rendered most mainstream varieties unable to adapt to the diversified demands of modern adzuki bean industrialization (Ha et al., 2025; Wu et al., 2025).

Previous studies have extensively explored individual quality traits of adzuki bean germplasms, yet several research gaps remain unresolved. Most existing investigations merely focus on isolated indicators such as agronomic performance, conventional nutrition or single antioxidant activity, lacking a holistic multi-dimensional evaluation framework integrating agronomy, processing property, nutritional quality and functional activity (Yin et al., 2025). Moreover, relevant studies are mostly restricted to a small number of germplasms or specific ecological regions, whereas systematic comparative assessments of widely cultivated adzuki bean varieties across major planting areas of China are still limited (Shi et al., 2026a). Given that adzuki bean comprehensive quality is a complex quantitative trait controlled by multiple interrelated factors, single index evaluation fails to objectively reflect the overall performance of cultivars. As a classical multivariate statistical method, principal component analysis (PCA) can effectively reduce dimensionality of complex phenotypic data and has been widely applied in crop quality comprehensive assessment (Chen et al., 2024; Shi et al., 2021).

In this study, 22 dominant adzuki bean varieties widely cultivated in China were selected as experimental materials. A broad set of indices related to agronomic traits, mineral nutrition, seed color, processing quality, nutritional components and functional activities were systematically determined, and 18 core indices covering five quality dimensions were selected for PCA. The present work aimed to: (1) characterize the phenotypic variation of major quality traits among mainstream adzuki bean cultivars; (2) clarify the phenotypic differentiation of tested varieties in each quality dimension; (3) establish a comprehensive quality evaluation system and screen promising candidate germplasms for targeted utilization. The findings are expected to provide a theoretical and practical basis for the screening of promising cultivars, genetic improvement of special-purpose cultivars, and diversified industrial exploitation of adzuki bean resources.

## Materials and methods

### Experimental site description

Field experiments were carried out from June 15 to October 15, 2025 at the Teaching Farm (38°51′N, 115°29′E) of Hebei Agricultural University, Baoding, China. The experimental site belongs to a warm temperate semi-humid continental monsoon climate, with an annual mean temperature of 12.3 °C, annual precipitation of 550 mm, and a frost-free period of approximately 189 days. The experimental soil was classified as loam, with winter wheat serving as the previous crop. Prior to sowing in 2025, the nutrient contents of the 0–20 cm topsoil layer were determined as follows: organic matter 15.13 g kg^−1^, total nitrogen 1.94 g kg^−1^, alkaline-hydrolyzable nitrogen 86.61 mg kg^−1^, available phosphorus 65.99 mg kg^−1^, and available potassium 186.44 mg kg^−1^. During the whole growth cycle of adzuki bean, field management including manual weeding and topdressing was implemented regularly. Pest and disease control was conducted via agricultural and biological measures without chemical pesticide application. Other cultivation practices were consistent with local conventional field production.

### Experimental design

The experiment was arranged in a randomized complete block design with 22 varieties (treatments) and three biological replicates for each treatment. Each experimental plot was 5 m in length and 2 m in width, covering an area of 10 m^2^, and a 1.5 m isolation belt was set between adjacent plots to eliminate marginal effects. Seeding was manually performed at a depth of 3–4 cm with two seeds per hill. Seedlings were thinned to one plant per hill at the three-leaf stage. The planting spacing was 0.3 m within rows and 0.5 m between rows, corresponding to a planting density of 66667 plants ha^−1^. A basal–topdressing fertilizer ratio of 5:5 was adopted. Specifically, 75 kg ha^−1^ pure nitrogen, 75 kg ha^−1^ P_2_O_5_, and 75 kg ha^−1^ K_2_O were applied as basal fertilizer before sowing. An additional 75 kg ha^−1^ nitrogen was topdressed with irrigation at the early flowering stage in late July. Adequate soil moisture was maintained throughout the growing season to avoid drought stress.

A total of 22 representative adzuki bean varieties originating from different ecological regions of China were selected as experimental materials, covering early, medium-early and late maturity types. Detailed information on the tested varieties is presented in **Table 1**.

**Table 1:** Adzuki bean varieties and breeding institutions.

Code	Variety	Maturity	Breeding institution	Origin latitude
A	Jihong 8	Medium-early	Hebei Academy of Agriculture and Forestry Sciences	38.1°N
B	Jihong 13	Medium-early	Hebei Academy of Agriculture and Forestry Sciences	38.1°N
C	Jihong 15	Medium-early	Hebei Academy of Agriculture and Forestry Sciences	38.1°N
D	Jihong 9218	Early	Hebei Academy of Agriculture and Forestry Sciences	38.1°N
E	Baohong 947	Early	Baoding Institute of Agricultural Sciences	38.8°N
F	Baohong 3992	Medium-early	Baoding Institute of Agricultural Sciences	38.8°N
G	Baohong 8824	Early	Baoding Institute of Agricultural Sciences	38.8°N
H	Henong 1	Early	Hebei Agricultural University	42.9°N
I	Henong 2	Early	Hebei Agricultural University	42.9°N
J	Henong 3	Early	Hebei Agricultural University	42.9°N
K	Baihong 2	Early	Baicheng Academy of Agricultural Sciences	45.6°N
L	Baihong 8	Early	Baicheng Academy of Agricultural Sciences	45.6°N
M	Baihong 9	Early	Baicheng Academy of Agricultural Sciences	45.6°N
N	Liaohong 08712	Early	Liaoning Academy of Agricultural Sciences	41.8°N
O	Liaohong 08713	Early	Liaoning Academy of Agricultural Sciences	41.8°N
P	Liaohong 08715	Early	Liaoning Academy of Agricultural Sciences	41.8°N
Q	Kenjianhong	Early	Heilongjiang Academy of Agricultural Sciences	43.3°N
R	Zhenzhuhong	Early	Heilongjiang Academy of Agricultural Sciences	43.3°N
S	Hongquan 2	Early	Heilongjiang Academy of Agricultural Sciences	43.3°N
T	Hongquan 3	Early	Heilongjiang Academy of Agricultural Sciences	43.3°N
U	Yuhong 1	Late	Chongqing Academy of Agricultural Sciences	29.5°N
V	Yuhong 2	Late	Chongqing Academy of Agricultural Sciences	29.5°N

### Determination items and methods

#### Grain yield and yield component determination

At physiological maturity, nine uniform plants were randomly selected from each plot to determine yield-related traits, including pod length, pod width, pods per plant, seeds per pod, and 100-seed weight. The actual grain yield per hectare was estimated based on these yield components: the grain weight per plant was first derived from pods per plant, seeds per pod and 100-seed weight, and then scaled up to hectare basis according to the actual planting density adopted in the field trial.

#### Determination of seed nitrogen, phosphorus and potassium contents

For all seed quality and biochemical traits, seeds harvested from the three replicate plots of each cultivar were thoroughly pooled into a single representative bulk sample. This operation was adopted to average out micro-environmental variation among plots and more accurately reflect the average quality performance of each cultivar under the tested conditions. Three subsamples (10 g each) were taken from the bulk sample as technical replicates for subsequent determination. All samples were deactivated at 105 °C for 30 min, dried to a constant weight at 80 °C, and ground to pass through a 100-mesh sieve. The digested solution was prepared using the H_2_SO_4_-H_2_O_2_ wet digestion method. All mineral element contents were expressed on a dry weight basis. Seed nitrogen and phosphorus concentrations were determined by flow injection analysis (Smartchem 200, Alliance, France), while potassium content was measured via flame spectrophotometry (ZA3000, ELE Instrument Co., Ltd., Japan).

#### Seed appearance quality determination

Fifty intact and uniformly sized seeds were randomly selected from the bulk sample of each cultivar for color measurement, with three technical replicates. The lightness (L*), redness (a*), and yellowness (b*) values were determined using an NR310 portable colorimeter under D65 standard illuminant with a 10° viewing angle and an 8 mm aperture. Hue angle (H°) and chroma (C*) were calculated by the following formulas:

$\text{Hue}=\arctan\left({\text{b}}^{*}/{\text{a}}^{*}\right)$.


$$\text{Chroma}=\sqrt{{\left({\text{a}}^{*}\right)}^{2}+{\left({\text{b}}^{*}\right)}^{2}}$$

Determination of processing quality characteristics.

All processing quality assays were performed using the bulk seed sample of each cultivar, with three technical replicates. The bean paste yield was measured according to a previously reported method (Dong, 1991). Briefly, a fixed mass of cleaned adzuki bean seeds was placed in an aluminum box, soaked in distilled water for 10 h at room temperature, and then steamed for 1 h. The steamed seeds were fully ground and sieved through a 60-mesh screen to separate bean paste. The wet bean paste was filtered and dried to a constant weight at 80 °C in a forced-draft oven. The bean paste yield was calculated as the ratio of dry bean paste weight to initial dry seed weight.

$\text{C}=\frac{\text{W}0}{\text{M}}$

where W_o_ is the dry weight of bean paste (g), and M is the dry weight of the initial adzuki bean sample (g).

Textural characteristics: Adzuki bean seeds were pulverized with a 300 W grinder and sieved through a 40-mesh screen to obtain fine powder. Approximately 50 g of powder was placed in a petri dish and mixed with distilled water at a solid–liquid ratio of 1:1.5 (w/v) to form a homogeneous paste. The paste was steamed in boiling water for 45 min and equilibrated at room temperature for 10 min prior to testing. A TMS-PRO texture analyzer (FTC Co., Ltd., USA) was employed with the following parameters: test speed 60 mm min^−1^, sample deformation 50%, trigger force 0.5 N. Each sample was measured in five technical replicates, and the maximum and minimum values were discarded before calculating the mean. Determined textural indices included hardness, cohesiveness, springiness, gumminess, and chewiness.

#### Measurement of nutritional and functional components

All nutritional and functional component determinations were conducted on the bulk seed sample of each cultivar, with three technical replicates for each assay. All results were expressed on a dry weight basis. Total starch, amylose and amylopectin: Total starch content was assayed using a modified anthrone-sulfuric acid colorimetric method (Liu et al., 2023). In brief, 0.2 g of dried seed powder was suspended in 2 mL ultrapure water and boiled for 15 min. After cooling, samples were extracted with 2 mL perchloric acid in a water bath for 15 min. The extraction procedure was repeated three times, and combined supernatants were diluted to 50 mL. A 2 mL aliquot was mixed with 5 mL anthrone reagent (0.2% w/v in 95% H_2_SO_4_) and incubated at 100 °C for 10 min. The absorbance was recorded at 630 nm, and total starch content was quantified using a glucose standard curve (*R^2^* > 0.999). Amylose and amylopectin contents were determined using commercial assay kits (Solarbio Biotechnology Co., Ltd., Beijing, China) in accordance with the manufacturer’s protocols. The amylose/amylopectin (AM/AP) ratio was calculated on a dry weight basis.

Soluble Sugar: Soluble sugar was determined via the anthrone colorimetric method (Li et al., 2022). Briefly, 0.2 g of seed powder was extracted three times with 20 mL absolute ethanol at 80 °C for 30 min each time. Combined filtrates were diluted to 100 mL. A 1 mL aliquot was evaporated to dryness, redissolved in 1 mL ultrapure water, and reacted with 5 mL anthrone reagent at 100 °C for 10 min. The absorbance was measured at 630 nm, and soluble sugar content was calculated using a sucrose standard curve.

Soluble Protein: Soluble protein content was determined by the Coomassie Brilliant Blue G-250 method (Bradford, 1976). A total of 0.1 g seed powder was homogenized with 5 mL distilled water and centrifuged at 8000×g for 10 min. Subsequently, 1 mL supernatant was mixed with 5 mL Coomassie G-250 staining solution, and the absorbance was measured at 595 nm. Soluble protein concentration was quantified using a bovine serum albumin standard curve (0–100 μg mL^−1^).

Total phenolics were determined using the modified Folin-Ciocalteu method (Niu et al., 2022). In brief, 0.2 g seed powder was extracted with 10 mL of 70% (v/v) aqueous methanol via ultrasonication at 40 °C for 30 min, followed by centrifugation at 5000×g for 10 min. A 0.2 mL aliquot of supernatant was mixed with 1.5 mL 10% (w/v) Na_2_CO_3_ solution and diluted to 10 mL with ultrapure water. The mixture was incubated at 35 °C for 30 min, and the absorbance was measured at 765 nm.

Total flavonoids were measured using the modified NaNO_2_-Al(NO_3_)_3_ colorimetric method (Dong et al., 2025). Seed powder was extracted with 70% (v/v) aqueous methanol following the same procedure as total phenol determination. A 1 mL extract aliquot was mixed with 1 mL 5% (w/v) NaNO_2_ solution and allowed to stand for 6 min. Then, 1 mL 10% (w/v) Al(NO_3_)_3_ solution was added and incubated for another 6 min. Finally, 4 mL 4% (w/v) NaOH solution was added, and the volume was adjusted to 10 mL. After 15 min of standing, the absorbance was determined at 510 nm.

Total isoflavone content was determined using a commercial assay kit (Suzhou Grace Biotechnology Co., Ltd., Suzhou, China). Briefly, 0.1 g dried seed powder was homogenized with 1 mL kit extraction solution and ultrasonicated for 10 min. The mixture was centrifuged at 12000×g for 10 min at room temperature, and the supernatant was discarded. The precipitation was re-extracted once using the same procedure and air-dried. Subsequently, 1.5 mL 70% ethanol was added, vortexed for 5 min, and ultrasonicated for 20 min. After centrifugation at 8000×g for 10 min, the supernatant was collected, and the absorbance was measured at 260 nm using an ultraviolet spectrophotometer.

Saponin content was determined with minor modifications to the method described previously (Chen and Chang, 2015). A total of 0.5 g seed powder was extracted with 10 mL 80% methanol in a constant-temperature water bath at 60 °C for 3 h. The extract was centrifuged at 12000×g for 20 min at 4 °C. A 0.1 mL aliquot of supernatant was mixed sequentially with 0.4 mL 80% methanol, 0.5 mL 8% vanillin–ethanol solution, and 5 mL 72% sulfuric acid solution. The mixture was incubated in a water bath at 60 °C for 10 min after ice-bath mixing, and the absorbance was measured at 544 nm after cooling. Saponin content was calculated using an oleanolic acid standard curve.

GABA content was quantified using a plant GABA detection kit (Gereis Biotechnology Co., Ltd., Suzhou, China) with a microplate reader. The determination was based on the color reaction of GABA with hypochlorite and phenol under alkaline conditions. The absorbance of the reaction mixture was measured at 645 nm, and GABA concentration was calculated according to the standard curve provided by the kit.

For bound isoflavone determination (Zhang et al., 2011), 0.1 g seed powder was mixed with 4 mL 80% (v/v) methanol solution, kept at room temperature for 2.5 h, and extracted in an 80 °C water bath for 14 h. The extract was centrifuged at 12000×g for 15 min, and the supernatant was filtered through a 0.45 μm membrane and stored at 4 °C prior to HPLC analysis. Determinations were performed on an Agilent 1200 HPLC system equipped with a Phenomenex C18 column. The chromatographic conditions were set as follows: column temperature 40 °C; mobile phase methanol–water (30:70, v/v); flow rate 1.0 mL min^−1^; detection wavelength 254 nm; injection volume 10 μL; run time 40–50 min per sample. Daidzin, glycitin, and genistin standards (Sigma-Aldrich, USA) were used for qualitative and quantitative analysis based on retention time and peak area. All samples were analyzed in triplicate.

For free daidzein and genistein determination (Guo et al., 2012), 1.0 g seed powder was extracted with 5 mL chromatographic-grade methanol via ultrasonication at room temperature for 1 h and kept stationary for 24 h. A 1.5 mL aliquot of supernatant was centrifuged at 12000×g for 20 min, filtered through a 0.45 μm membrane, and analyzed by HPLC. An Agilent 1200 system with a C18 reversed-phase column was used at 25 °C. Mobile phase A was chromatographic ethanol, and mobile phase B was 0.1% (v/v) acetic acid solution (pH 3.22). Isocratic elution was performed at A:B = 40:60 (v/v) with a flow rate of 1.0 mL min^−1^, detection wavelength 254 nm, and injection volume 10 μL. Daidzein and genistein standards (Sigma-Aldrich, USA) were used for quantification, with three replicate injections for each sample.

#### Antioxidant capacity determination

All antioxidant activity assays were performed using the same bulk sample extract, with three technical replicates. Ferric Reducing Antioxidant Power (FRAP) Assay: The method of Sochor et al. was used with minor modifications (Sochor et al., 2010). Briefly, a 0.1 mL aliquot of the same extract used for total phenolics and flavonoids determination was mixed thoroughly with 3 mL of FRAP working solution and 0.3 mL of ultrapure water. After reacting for 5 min at room temperature, the absorbance of the mixture was measured at 593 nm. The FRAP value was quantified using a ferrous sulfate (FeSO_4_) standard curve and expressed as μmol g^−1^ on a dry weight basis, while DPPH·, ABTS^+^· and ·OH scavenging activities were expressed as radical scavenging rate (%).

1,1-Diphenyl-2-picrylhydrazyl (DPPH·) Radical Scavenging Activity: The method of Hu et al. was used with minor modifications (Hu et al., 2004). Briefly, 1 mL of the sample extract was mixed with 4 mL of 0.25 mmol·L^−1^ DPPH methanolic solution. The mixture was incubated in the dark at 37 °C for 30 min, then centrifuged at 4000 r·min^−1^ for 10 min. The absorbance of the supernatant was measured at 510 nm.


$$\text{DPPH}\cdot \text{Radical Scavenging Activity}=\left(1-\frac{\text{A}1-\text{A}2}{\text{A}0}\right)\times 100\%$$

Where: A_1_ is the absorbance of the sample supernatant; A_o_ is the absorbance of the blank control (60% ethanol instead of the sample extract); A_2_ is the absorbance of the sample background control (1 mL sample extract mixed with 4 mL anhydrous ethanol under the same reaction conditions).

2,2’-Azino-bis(3-ethylbenzothiazoline-6-sulfonic Acid) Diammonium Salt (ABTS^+^·) Radical Scavenging Activity: The method of Zhou et al. was used with minor modifications (Zhou et al., 2004).

)Briefly, 0.1 mL of the sample extract was mixed with 3.9 mL of ABTS working solution in a centrifuge tube. After accurate reaction for 6 min at room temperature in the dark, the absorbance of the mixture was measured at 734 nm.


$${\text{ABTS}}^{+}\cdot \text{Radical Scavenging Activity}=\left(1-\frac{A1}{A0}\right)\times 100\%$$

Where: A_1_ is the absorbance of the mixture of sample extract and ABTS working solution after reaction; A_o_ is the absorbance of the blank control (60% ethanol instead of the sample extract).

Hydroxyl Radical (·OH) Scavenging Activity: The method of Wang et al. was used with minor modifications (Feng et al., 2022). Briefly, 2 mL of 9 mmol L^−1^ FeSO_4_ solution, 2 mL of sample extract, and 2 mL of H_2_O_2_ solution were sequentially added into a centrifuge tube, mixed thoroughly, and left to stand at room temperature for 10 min. Subsequently, 2 mL of 9 mmol·L^−1^ salicylic acid-ethanol solution was added to the mixture, which was then incubated in a water bath at 25 °C for 30 min after mixing. The absorbance of the reaction solution was measured at 510 nm and recorded as A_1_. Under the same reaction conditions, the absorbance of the blank control (60% ethanol instead of the sample extract) was measured and recorded as A_o_; the absorbance of the background control (anhydrous ethanol instead of salicylic acid-ethanol solution) was measured and recorded as A_2_.


$$\cdot \text{OH Radical Scavenging Activity}=\left(1-\frac{\text{A}1-\text{A}2}{\text{A}0}\right)\times 100\%$$

Where: A_1_ is the absorbance of the mixture of sample extract and all reagents after reaction; A_o_ is the absorbance of the blank control (60% ethanol instead of the sample extract); A_2_ is the absorbance of the background control (anhydrous ethanol instead of salicylic acid-ethanol solution).

### Statistical analysis

Raw data were organized and preliminarily calculated using Microsoft Excel 2020 (Microsoft Corp., Redmond, WA, USA). For agronomic and yield traits determined at the individual plot level, analysis of variance (ANOVA) was performed based on the randomized complete block design, with cultivar set as a fixed effect and block as a random effect. For quality, processing, nutritional and functional traits determined from bulk samples, one-way ANOVA was performed based on three technical replicates to compare phenotypic differences among cultivars. Duncan’s multiple range test was adopted for *post-hoc* multiple comparisons at a significance level of *P* < 0.05. Principal component analysis (PCA) was conducted on 18 core quality indicators to reduce data dimensionality, calculate comprehensive scores, and achieve multi-variety comprehensive quality evaluation. All statistical analyses were performed using DPS v14.10 (Hangzhou Ruifeng Information Technology Co., Ltd., Hangzhou, China). Data visualization was generated using GraphPad Prism 8.4.3 (GraphPad Software, LLC., San Diego, CA, USA).

## Results

### Differences in agronomic traits and yield

Analysis of variance based on the randomized complete block design showed that all six agronomic and yield-related traits exhibited significant differences among the 22 adzuki bean varieties (*P* < 0.05). The coefficient of variation (CV) of the measured traits ranged from 11.76% to 90.90%. Pods per plant presented the highest CV value of 90.90%, followed by seeds per pod (22.79%) and pod width (20.86%), while 100-seed weight (11.94%) and grain yield (11.76%) showed relatively low phenotypic variation (**Table 2**). Pod length varied from 6.68 cm to 8.79 cm, with Baihong 2 (K) recording the maximum value and Zhenzhuhong (R) the minimum. Pod width ranged between 5.29 cm and 6.43 cm, with Baihong 2 (K) having the widest pods and Liaohong 08712 (N) the narrowest. The number of pods per plant varied from 16.67 to 31.67, with Henong 3 (J) producing the most pods and Yuhong 2 (V) the fewest. Seeds per pod ranged from 5.93 to 8.94, and the maximum value was observed in Hongquan 2 (S), whereas Henong 1 (H) had the lowest value. The 100-seed weight fluctuated from 6.52 g to 13.64 g, with Jihong 13 (B) showing the significantly highest value and Henong 3 (J) the lowest. Grain yield differed significantly across varieties, ranging from 833.49 kg ha^−1^ to 1540.44 kg ha^−1^. Jihong 9218 (D) yielded the highest production, followed by Jihong 13 (B, 1385.46 kg ha^−1^) and Henong 2 (I, 1326.28 kg ha^−1^), while Yuhong 1 (U) obtained the lowest yield. Phenotypic characterization of yield components showed that Jihong 9218 was characterized by a high pod number per plant, and Jihong 13 performed with a large 100-seed weight. By contrast, Henong 3 possessed the greatest pod number but exhibited a low 100-seed weight, resulting in a moderate-to-low grain yield.

**Table 2:** Index of adzuki bean output.

Code	Pod length (cm)	Pod width (cm)	Pods per plant	Seeds per pod	100-seed weight (g)	Yield (kg ha^-1^)
A	8.57 ± 0.03b	6.25 ± 0.05c	23.00 ± 1.00fg	8.42 ± 0.04b	10.50 ± 0.03i	1302.46 ± 3.12d
B	8.49 ± 0.05b	6.37 ± 0.09ab	20.33 ± 0.58ij	7.80 ± 0.08d	13.64 ± 0.13a	1385.46 ± 13.1b
C	7.48 ± 0.12gh	6.27 ± 0.04c	20.33 ± 0.58ij	6.97 ± 0.03h	11.52 ± 0.05e	1045.92 ± 4.72h
D	7.77 ± 0.02f	5.64 ± 0.03hi	29.67 ± 0.58b	7.11 ± 0.13g	11.40 ± 0.05f	1540.44 ± 6.76a
E	7.46 ± 0.06ghi	5.51 ± 0.03j	20.00 ± 1.00ijk	6.82 ± 0.07i	10.34 ± 0.10j	903.38 ± 8.30n
F	7.36 ± 0.05i	5.52 ± 0.06j	19.67 ± 1.15jk	7.45 ± 0.12f	10.85 ± 0.05gh	1017.15 ± 4.23i
G	8.14 ± 0.07cd	5.55 ± 0.08ij	17.67 ± 1.15lm	7.60 ± 0.09e	10.96 ± 0.04g	941.57 ± 3.25l
H	6.95 ± 0.02j	6.31 ± 0.02bc	28.00 ± 1.00c	5.93 ± 0.09l	9.97 ± 0.07k	1059.76 ± 7.46g
I	7.55 ± 0.03g	5.35 ± 0.1kl	27.00 ± 1.00cd	7.09 ± 0.03gh	10.82 ± 0.05h	1326.28 ± 6.49c
J	7.47 ± 0.05gh	5.41 ± 0.06k	31.67 ± 1.15a	7.10 ± 0.03gh	6.52 ± 0.03p	938.21 ± 4.98lm
K	8.79 ± 0.02a	6.43 ± 0.04a	17.33 ± 1.53lm	8.07 ± 0.03c	10.36 ± 0.14j	927.63 ± 12.58m
L	7.52 ± 0.12g	5.66 ± 0.07h	18.67 ± 1.15kl	7.86 ± 0.08d	10.01 ± 0.03k	940.42 ± 2.49l
M	7.05 ± 0.07j	5.92 ± 0.02de	19.67 ± 0.58jk	8.39 ± 0.05b	9.42 ± 0.08m	995.62 ± 8.54k
N	8.18 ± 0.01cd	5.29 ± 0.08l	22.33 ± 0.58gh	6.80 ± 0.11i	10.40 ± 0.03ij	1011.83 ± 2.92ij
O	6.80 ± 0.06k	5.82 ± 0.08ef	24.33 ± 0.58ef	6.73 ± 0.04i	8.66 ± 0.06o	908.10 ± 5.84n
P	7.47 ± 0.10ghi	5.88 ± 0.06de	20.67 ± 0.58ij	6.55 ± 0.12j	12.88 ± 0.10b	1116.13 ± 8.23e
Q	7.38 ± 0.05hi	5.76 ± 0.04fg	24.67 ± 0.58e	6.06 ± 0.05l	10.51 ± 0.01i	1005.41 ± 0.96jk
R	6.68 ± 0.06l	5.93 ± 0.08d	26.33 ± 0.58d	7.01 ± 0.12gh	8.94 ± 0.02n	1056.21 ± 2.05gh
S	8.24 ± 0.08c	6.27 ± 0.03c	18.00 ± 2.00lm	8.94 ± 0.07a	9.83 ± 0.04l	1012.54 ± 4.16ij
T	8.12 ± 0.08d	5.67 ± 0.03gh	21.33 ± 0.58hi	6.30 ± 0.13k	12.82 ± 0.06b	1102.99 ± 4.74f
U	7.47 ± 0.07ghi	5.39 ± 0.09k	17.33 ± 1.53lm	6.35 ± 0.03k	11.82 ± 0.04d	833.49 ± 2.54o
V	7.89 ± 0.08e	5.67 ± 0.06gh	16.67 ± 0.58m	7.11 ± 0.10g	12.23 ± 0.09c	927.73 ± 7.05m
CV	16.62%	20.86%	90.90%	22.79%	11.94%	11.76%

Data are presented as mean ± standard deviation of three biological replicates (n = 3). Different lowercase letters in the same column indicate significant differences among cultivars at *P* < 0.05 according to Duncan’s multiple range test (RCBD-ANOVA).

### Differences in mineral elements and seed color

**Figure 1:**
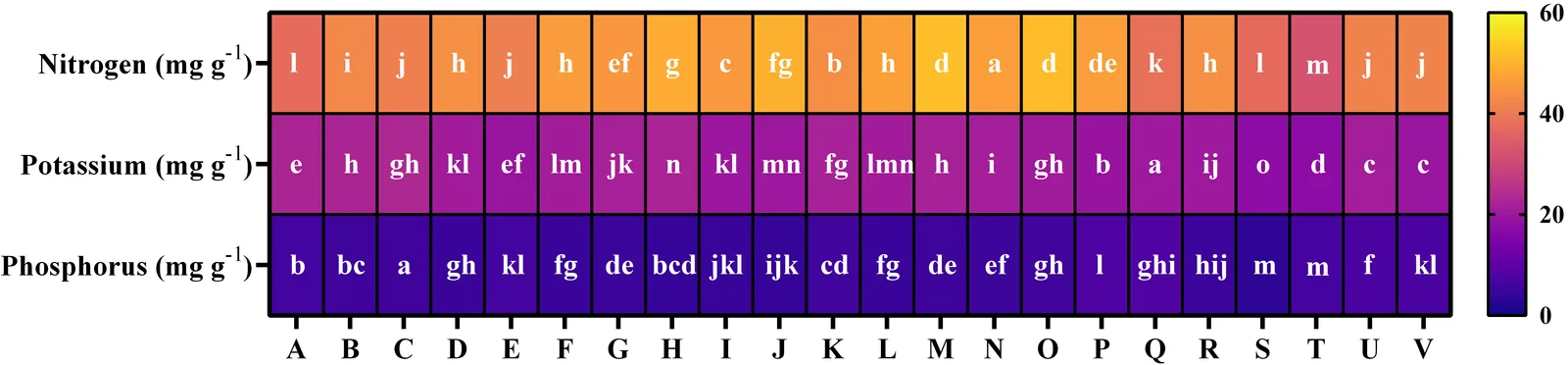
Heatmap showing the variations in seed nitrogen, phosphorus and potassium contents among 22 adzuki bean varieties. Data are presented as the mean of three technical replicates (n = 3). Different lowercase letters indicate significant differences at *P* < 0.05 according to Duncan’s multiple range test. Variety codes A-V correspond to those listed in **Table 1**. The color gradient from blue to yellow represents increasing values of the corresponding trait.

**Figure 2:**
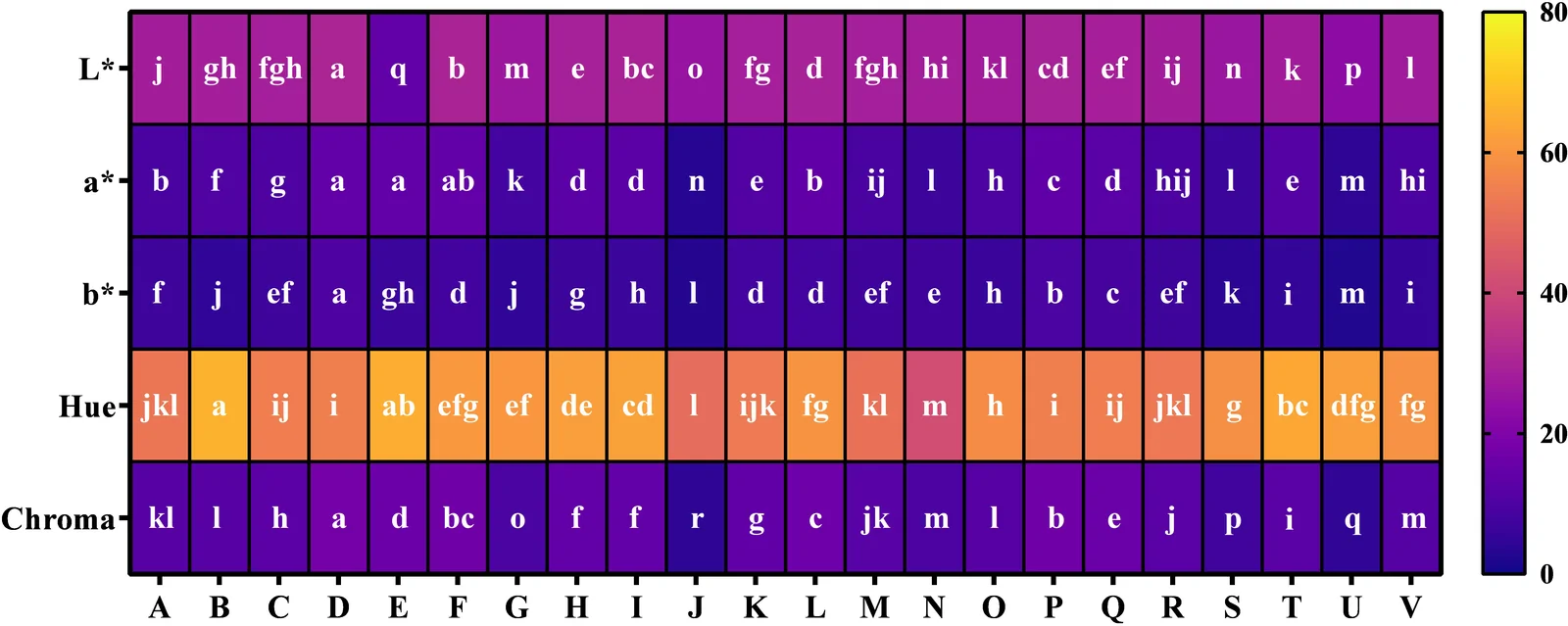
Heatmap showing the variations in seed color parameters (L*, a*, b*, hue and chroma) among 22 adzuki bean varieties. Data are presented as the mean of three technical replicates (n = 3). Different lowercase letters indicate significant differences at *P* < 0.05 according to Duncan’s multiple range test. Variety codes A-V correspond to those listed in **Table 1**. The color gradient from blue to yellow represents increasing values of the corresponding trait.

Significant differences in seed mineral nutrient contents and seed color parameters were observed among the tested varieties under the analytical conditions of this study (*P* < 0.05). The CV values of all measured traits varied from 10.66% to 58.57%. Phosphorus content had the highest CV of 58.57%, followed by yellowness (b*, 51.86%) and potassium content (33.54%), while lightness (L*) showed the lowest variation (10.66%). Seed nitrogen content on a dry weight basis ranged ranged from 32.15 mg g^−1^ to 51.44 mg g^−1^, with Baihong 9 and Liaohong 08713 presenting the maximum values and Hongquan 3 the minimum. Phosphorus content varied from 3.53 mg g^−1^ to 8.17 mg g^−1^, with Kenjianhong and Hongquan 2 showing the highest and lowest values, respectively. Potassium content fluctuated between 17.51 mg g^−1^ and 23.42 mg g^−1^, and the maximum was recorded in Jihong 15, while Hongquan 3 had the lowest potassium concentration (**Figure 1**; Attached **Table 1**). For seed color parameters, L* values ranged from 14.68 to 30.46; Jihong 9218 had the brightest seed coat, and Baohong 947 was the darkest. The a* value varied from 3.69 to 14.59, with Jihong 9218 and Baohong 947 having the highest redness and Henong 3 the lowest. The b* value ranged from 2.52 to 10.18, with Jihong 9218 and Yuhong 1 representing the maximum and minimum, respectively. Hue angle varied from 42.06 to 66.50, with Jihong 13 having the largest value and Liaohong 08712 the smallest. Chroma ranged from 4.73 to 17.79, and Jihong 9218 exhibited the highest chroma, while Henong 3 had the dullest seed color (**Figure 2**; Attached **Table 1**).

### Differences in processing quality

All six processing quality traits showed significant differences among the 22 adzuki bean varieties under the tested conditions (*P* < 0.05). The CV values ranged from 16.84% to 57.23%. Springiness showed the highest variation (57.23%), followed by cohesiveness (32.09%) and chewiness (30.15%), whereas bean paste yield had the lowest CV (16.84%) (**Table 3**). Seed hardness varied from 61.84 N to 205.60 N; Liaohong 08715 (P) had the maximum hardness, and Baihong 2 (K) had the minimum. Cohesiveness ranged from 0.08 to 0.27, with Baohong 3992 (F) recording the highest value and Yuhong 2 (V) the lowest. Springiness fluctuated from 0.56 mm to 2.60 mm, with Baihong 9 (M) and Yuhong 2 (V) representing the maximum and minimum, respectively. Gumminess varied between 5.55 N and 51.06 N; Baohong 3992 (F) had the highest gumminess, and Baihong 2 (K) the lowest. Chewiness ranged from 3.27 mJ to 96.28 mJ, with Baohong 3992 (F) showing the highest value and Yuhong 2 (V) the lowest. As the core processing index, bean paste yield ranged from 0.43 to 0.70, with Baihong 2 (K) being significantly higher than all other varieties and Henong 3 (J) the lowest.

**Table 3:** Processing quality parameters of 22 adzuki bean varieties.

Code	Hardness (N)	cohesiveness (Ratio)	Springiness (mm)	Gumminess (N)	Chewiness (mJ)	Bean paste yield (Ratio)
A	117.96 ± 1.39l	0.16 ± 0.01ef	2.27 ± 0.05ef	17.95 ± 0.36m	41.73 ± 0.53j	0.57 ± 0.01ij
B	133.05 ± 0.60j	0.14 ± 0.01i	2.44 ± 0.03bc	20.82 ± 0.47k	45.24 ± 0.82i	0.51 ± 0.01o
C	167.69 ± 0.42c	0.16 ± 0.01ef	2.38 ± 0.04cd	27.16 ± 0.21g	65.09 ± 0.45c	0.64 ± 0.01d
D	148.00 ± 0.96g	0.16 ± 0.01e	2.04 ± 0.07h	28.07 ± 0.10f	58.52 ± 0.56e	0.52 ± 0.01n
E	179.50 ± 2.31b	0.13 ± 0.01j	2.19 ± 0.05f	28.00 ± 0.09f	61.99 ± 0.35d	0.53 ± 0.01m
F	159.53 ± 1.32e	0.27 ± 0.01a	2.19 ± 0.03fg	51.06 ± 0.37a	96.28 ± 1.02a	0.59 ± 0.01g
G	157.76 ± 0.99e	0.15 ± 0.01gh	2.31 ± 0.07de	23.40 ± 0.46j	54.56 ± 0.33g	0.61 ± 0.01f
H	163.93 ± 2.37d	0.17 ± 0.01d	1.88 ± 0.07i	28.99 ± 0.02e	57.05 ± 0.34f	0.58 ± 0.01h
I	180.99 ± 1.79b	0.10 ± 0.01l	2.03 ± 0.01h	45.44 ± 0.18b	54.66 ± 0.35g	0.47 ± 0.01p
J	127.77 ± 0.61k	0.10 ± 0.01l	1.38 ± 0.02k	13.65 ± 0.20p	19.32 ± 0.92m	0.43 ± 0.01q
K	61.84 ± 1.11o	0.09 ± 0.01m	0.66 ± 0.09m	5.55 ± 0.19r	4.35 ± 0.54o	0.70 ± 0.01a
L	141.18 ± 1.44h	0.14 ± 0.01i	2.28 ± 0.09ef	20.06 ± 0.35l	47.06 ± 0.93h	0.67 ± 0.01b
M	136.92 ± 1.34i	0.17 ± 0.01d	2.60 ± 0.07a	24.39 ± 0.48i	62.48 ± 0.91d	0.55 ± 0.01l
N	148.87 ± 3.03fg	0.16 ± 0.01e	2.01 ± 0.06h	28.13 ± 0.07f	27.88 ± 0.25k	0.57 ± 0.01i
O	138.14 ± 0.55i	0.11 ± 0.01k	2.38 ± 0.03cd	16.63 ± 0.34n	16.56 ± 1.05n	0.64 ± 0.01d
P	205.60 ± 0.17a	0.15 ± 0.01h	2.10 ± 0.03gh	28.97 ± 0.25e	28.54 ± 0.31k	0.51 ± 0.01o
Q	168.41 ± 2.27c	0.19 ± 0.01c	2.41 ± 0.08bc	37.94 ± 0.21c	65.67 ± 0.83c	0.57 ± 0.01jk
R	150.85 ± 1.83f	0.15 ± 0.01fg	2.50 ± 0.11b	25.11 ± 0.35h	62.42 ± 0.79d	0.63 ± 0.01e
S	125.73 ± 1.50k	0.21 ± 0.01b	2.46 ± 0.04bc	31.81 ± 0.28d	67.17 ± 0.22b	0.63 ± 0.01e
T	93.03 ± 1.01m	0.13 ± 0.01j	1.62 ± 0.06j	17.16 ± 0.45n	21.79 ± 1.13l	0.66 ± 0.01c
U	141.31 ± 3.21h	0.15 ± 0.01gh	0.92 ± 0.04l	11.56 ± 0.12q	18.91 ± 0.44m	0.56 ± 0.01k
V	70.70 ± 0.83n	0.08 ± 0.01n	0.56 ± 0.01n	15.81 ± 0.78o	3.27 ± 0.39o	0.46 ± 0.01p
CV	21.89%	32.09%	57.23%	25.48%	30.15%	16.84%

Data are presented as mean ± standard deviation of three technical replicates. Different lowercase letters in the same column indicate significant differences among cultivars at *P* < 0.05 according to Duncan’s multiple range test.

### Differences in nutritional components

**Figure 3:**
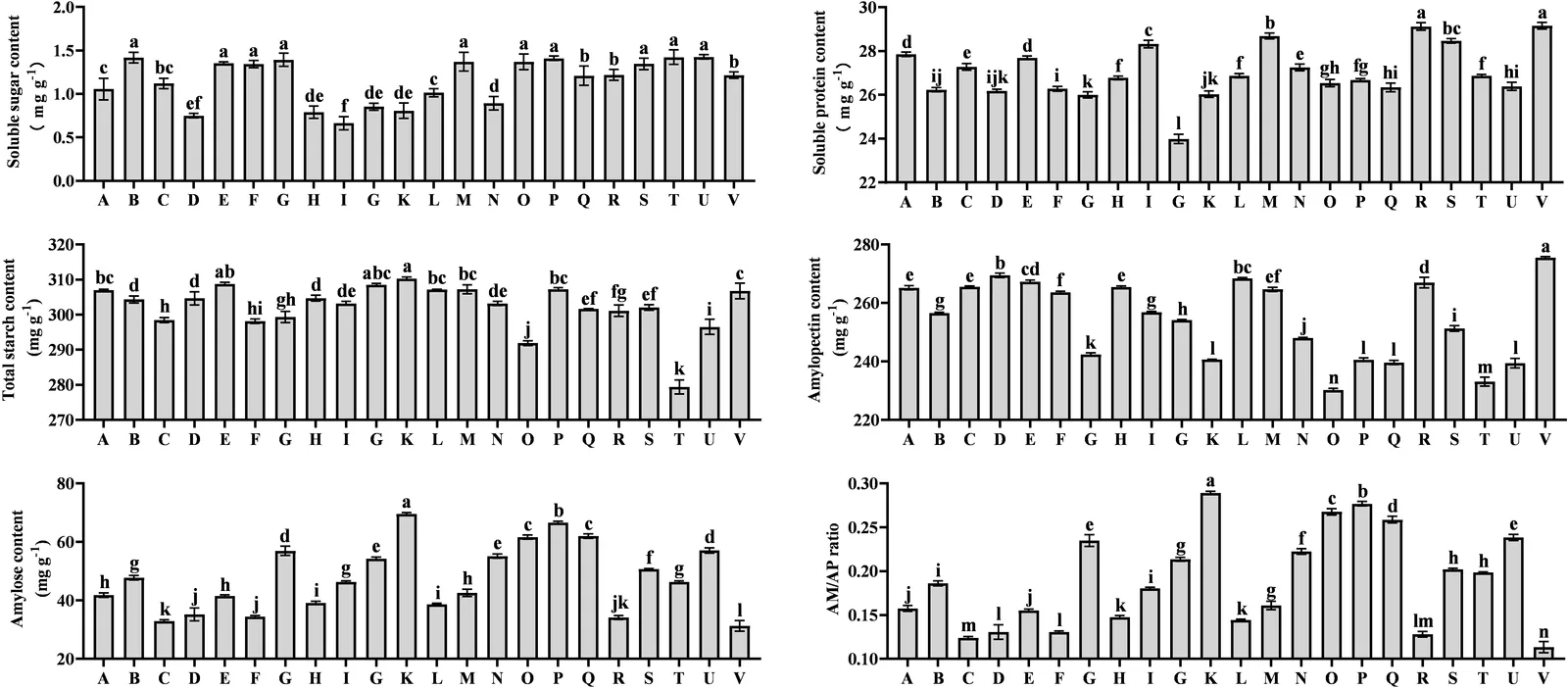
Soluble sugar, protein, starch components and amylose/amylopectin (AM/AP) ratio in seeds of 22 adzuki bean varieties. Error bars represent standard deviation of three technical replicates (n = 3). Different lowercase letters indicate significant differences at *P* < 0.05 according to Duncan’s multiple range test. Variety codes A-V correspond to those listed in **Table 1**.

Significant differences in six nutritional component indicators were observed across all varieties under the tested conditions (*P* < 0.05), with CV values ranging from 5.40% to 122.01%. Soluble sugar content exhibited the highest CV of 122.01%, followed by amylose/amylopectin (AM/AP) ratio (35.41%) and amylose content (34.91%) (**Figure 3**). By comparison, amylopectin (5.40%) and total starch (6.84%) presented relatively low phenotypic variation. Soluble sugar content ranged from 0.66 mg g^−1^ to 1.43 mg g^−1^; Yuhong 1, Hongquan 3 and Jihong 13 had higher soluble sugar levels, while Henong 2 had the lowest value. Soluble protein content varied from 23.99 mg g^−1^ to 29.15 mg g^−1^, with Yuhong 2 and Zhenzhuhong showing the highest values and Henong 3 the lowest. Total starch content on a dry weight basis fluctuated from 279.43 mg g^−1^ to 310.28 mg g^−1^; Baihong 2 had the maximum starch content, and Hongquan 3 the minimum. Amylopectin content ranged from 230.26 mg g^−1^ to 275.51 mg g^−1^, with Yuhong 2 and Liaohong 08713 representing the highest and lowest values, respectively. Amylose content varied from 31.27 mg g^−1^ to 69.62 mg g^−1^; Baihong 2 had the highest amylose content, and Yuhong 2 the lowest. The AM/AP ratio ranged from 0.11 to 0.29, and Baihong 2 showed a significantly higher ratio than other varieties, while Yuhong 2 had the lowest value.

### Differences in functional components and antioxidant activities

**Figure 4:**
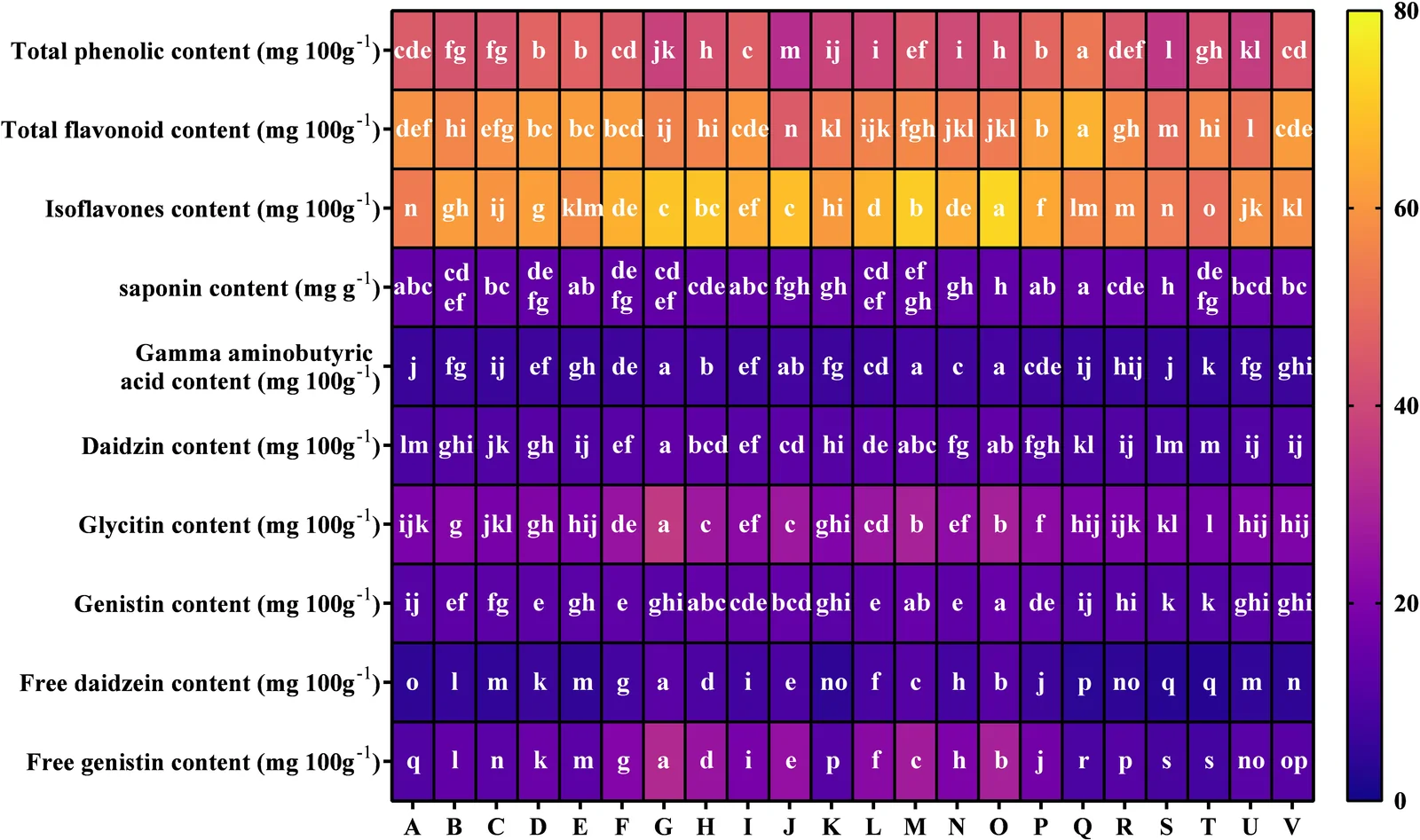
Heatmap showing the variations in phenolic, flavonoid, isoflavone, saponin and GABA contents among 22 adzuki bean varieties. Data are presented as the mean of three technical replicates (n = 3). Different lowercase letters indicate significant differences at *P* < 0.05 according to Duncan’s multiple range test. Variety codes A-V correspond to those listed in **Table 1**. The color gradient from blue to yellow represents increasing values of the corresponding trait.

**Figure 5:**
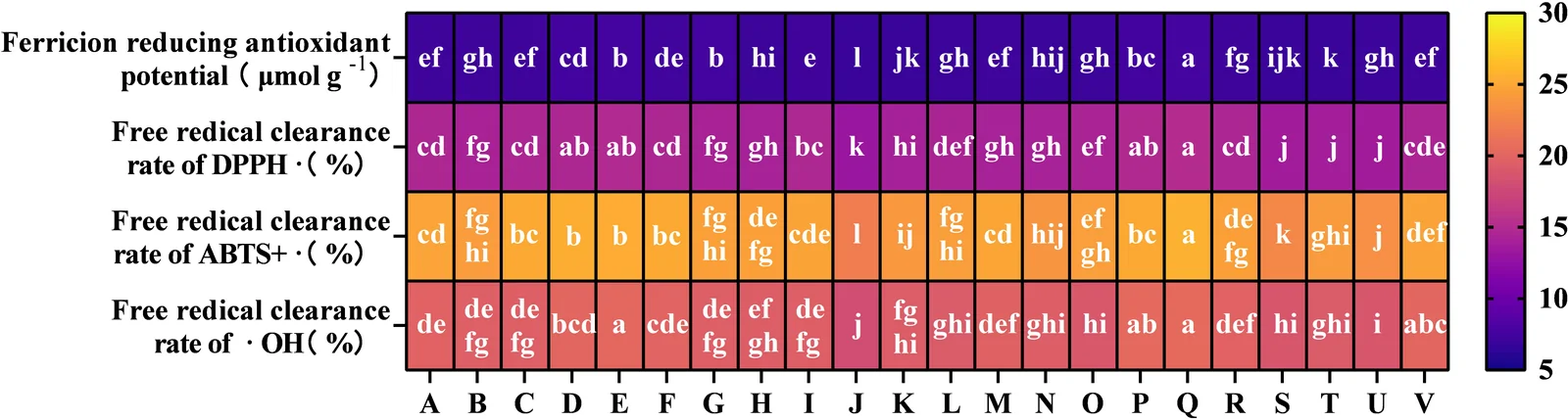
Heatmap showing the variations in FRAP, DPPH, ABTS+· and ·OH radical scavenging activities among 22 adzuki bean varieties. Data are presented as the mean of three technical replicates (n = 3). Different lowercase letters indicate significant differences at *P* < 0.05 according to Duncan’s multiple range test. Variety codes A-V correspond to those listed in **Table 1**. The color gradient from blue to yellow represents increasing values of the corresponding trait.

Fourteen functional and antioxidant indicators showed significant differences among varieties under the tested conditions (*P* < 0.05), with CV values distributed between 11.02% and 77.84%. Glycitin content had the highest CV (77.84%), followed by genistin (70.61%), free daidzein (69.00%) and GABA content (46.52%). DPPH radical scavenging activity showed the lowest variation (11.02%) (**Figures 4**, **5**; Attached **Table 2** & Attached **Table 3**). Total phenolic content (TPC) ranged from 32.60 mg 100g^−1^ to 52.37 mg 100g^−1^, with Kenjianhong having the highest value and Henong 3 the lowest. Total flavonoid content (TFC) varied from 45.67 mg 100g^−1^ to 65.59 mg 100g^−1^, and the maximum was also observed in Kenjianhong. Total isoflavone content (TIC) fluctuated from 50.91 mg 100g^−1^ to 73.11 mg 100g^−1^, with Liaohong 08713 recording the highest value and Hongquan 3 the lowest. Saponin content ranged from 13.56 mg g^−1^ to 14.97 mg g^−1^, with Kenjianhong and Hongquan 3 representing the maximum and minimum, respectively. GABA content varied from 6.20 mg 100g^−1^ to 8.90 mg 100g^−1^, and Baohong 8824, Baihong 9 and Liaohong 08713 had significantly higher GABA levels than other varieties. For isoflavone monomers, daidzin content ranged from 1.39 mg 100g^−1^ to 8.95 mg 100g^−1^; Baohong 8824 had the highest value, and Hongquan 3 the lowest. Glycitin content varied from 17.43 mg 100g^−1^ to 36.05 mg 100g^−1^, with Baohong 8824 as the maximum. Genistin content ranged from 10.76 mg 100g^−1^ to 15.25 mg 100g^−1^, with Liaohong 08713 recording the highest value. Free daidzein content fluctuated from 3.26 mg 100g^−1^ to 12.25 mg 100g^−1^, and Baohong 8824 had the highest concentration. Free genistein content ranged from 8.74 mg 100g^−1^ to 31.45 mg 100g^−1^, with Baohong 8824 being significantly higher than other varieties. In terms of antioxidant capacity, FRAP values ranged from 6.91 μmol g^−1^ to 7.67 μmol g^−1^; Kenjianhong had the highest activity and Henong 3 the lowest. DPPH scavenging rate varied from 13.15% to 15.09%, with Kenjianhong presenting the maximum value. ABTS^+^· scavenging rate ranged from 21.98% to 26.01%, and the highest was recorded in Kenjianhong. Hydroxyl radical scavenging activity varied from 18.06% to 20.81%, with Baohong 947 having the highest capacity and Henong 3 the lowest.

### Comprehensive evaluation based on principal component analysis

Principal component analysis (PCA) was performed using 18 core quality indicators to evaluate the comprehensive performance of 22 adzuki bean varieties (**Tables 4**–**6**). The eigenvalues of the first six principal components were all greater than 1, with a cumulative variance contribution of 79.14%, which could reflect the majority of information from the original dataset. The first principal component (PC1) accounted for 27.79% of the total variance and was positively correlated with FRAP, DPPH scavenging rate, seed redness (a*), hardness and total phenolic content. The second principal component (PC2) explained 16.33% of the total variance and was positively associated with 100-seed weight and pod length, while negatively correlated with total isoflavone content. Comprehensive scores of each variety were calculated based on the variance contribution of each principal component and then ranked. Baihong 2 (K) obtained the highest comprehensive score of 1.342, ranking first. Jihong 8 (A, 1.071) and Jihong 13 (B, 0.979) ranked second and third, respectively. Hongquan 2 (S) and Baohong 8824 (G) occupied the fourth and fifth positions. Zhenzhuhong (R) had the lowest comprehensive score of −1.188 and ranked last among all tested varieties.

**Table 4A:** Explanation of the total variance among the eighteen traits.

Initial eigenvalue	PC1	PC2	PC3	PC4	PC5	PC6	PC7	PC8	PC9
Eigenvalue	5.002	2.939	1.886	1.742	1.592	1.084	0.868	0.727	0.626
Contribution rate (%)	27.791	16.325	10.478	9.680	8.843	6.024	4.823	4.036	3.475
Cumulative contribution rate (%)	27.791	44.116	54.594	64.274	73.117	79.142	83.965	88.001	91.476

**Table 4B:** Explanation of the total variance among the eighteen traits.

Initial eigenvalue	PC10	PC11	PC12	PC13	PC14	PC15	PC16	PC17	PC18
Eigenvalue	0.524	0.319	0.197	0.171	0.115	0.098	0.072	0.023	0.015
Contribution rate (%)	2.910	1.772	1.093	0.951	0.639	0.546	0.402	0.127	0.084
Cumulative contribution rate (%)	94.386	96.158	97.251	98.203	98.841	99.387	99.789	99.916	100.000

**Table 5:** Eigenvectors of the first six principal components derived from eighteen quality traits.

Index	PC1	PC2	PC3	PC4	PC5	PC6
Pod length	-0.015	0.328	0.039	-0.108	-0.195	0.243
Pod width	-0.025	0.016	0.220	0.202	0.059	0.105
Pods per plant	-0.093	-0.166	0.277	-0.261	0.077	-0.179
Seeds per pod	-0.032	-0.002	0.016	0.007	-0.004	0.566
100-seed weight	0.106	0.343	-0.034	-0.062	-0.085	-0.052
Yield	0.004	0.135	0.293	-0.334	0.004	0.143
L* value	-0.011	-0.047	0.416	0.065	-0.075	-0.020
a* value	0.177	-0.007	0.092	0.113	-0.016	-0.047
b* value	0.154	-0.058	0.210	0.182	-0.046	-0.101
Hardness	0.175	-0.150	-0.150	-0.113	-0.168	0.186
Chewiness	0.122	-0.191	-0.076	-0.033	0.055	0.423
Sand yield	0.037	-0.094	0.070	0.496	-0.018	-0.030
Protein content	-0.03	0.003	-0.066	0.099	0.475	-0.084
AM/AP ratio	0.066	0.087	-0.041	0.120	-0.460	-0.052
Phenolic content	0.161	0.088	0.026	-0.014	0.088	-0.176
Isoflavone content	0.045	-0.265	0.053	0.025	-0.284	0.174
FRAP	0.236	0.051	-0.211	-0.062	-0.123	0.161
DPPH	0.200	0.040	-0.011	0.029	0.011	-0.036

Values represent the loading coefficients of each trait on the corresponding principal component.

**Table 6:** Comprehensive scores and rankings of the tested varieties calculated from the first six principal components.

vCode	Y1	Y2	Y3	Y4	Y5	Y6	F	Ranking
A	2.018	0.175	1.775	0.555	-1.114	1.935	1.071	2
B	1.845	0.160	1.623	0.507	-1.019	1.768	0.979	3
C	-0.438	-0.038	-0.385	-0.120	0.242	-0.420	-0.232	12
D	0.225	0.020	0.198	0.062	-0.124	0.216	0.119	7
E	-0.483	-0.042	-0.425	-0.133	0.267	-0.463	-0.256	16
F	0.025	0.002	0.022	0.007	-0.014	0.024	0.013	8
G	1.046	0.091	0.920	0.288	-0.578	1.003	0.555	5
H	-1.636	-0.142	-1.439	-0.450	0.903	-1.568	-0.868	20
I	-0.280	-0.024	-0.246	-0.077	0.154	-0.268	-0.148	9
J	-0.453	-0.039	-0.398	-0.125	0.250	-0.434	-0.240	13
K	2.531	0.220	2.226	0.696	-1.397	2.426	1.342	1
L	-0.355	-0.031	-0.312	-0.098	0.196	-0.340	-0.188	10
M	-1.418	-0.123	-1.247	-0.390	0.783	-1.359	-0.752	19
N	-1.137	0.099	1.000	0.312	-0.628	1.090	-0.195	11
O	-1.975	-0.172	-1.737	-0.543	1.090	-1.893	-1.048	21
P	-0.468	-0.041	-0.412	-0.129	0.258	-0.449	-0.248	14
Q	-0.664	-0.058	-0.584	-0.183	0.367	-0.637	-0.352	18
R	-2.239	-0.195	-1.969	-0.615	1.236	-2.146	-1.188	22
S	1.280	0.111	1.126	0.352	-0.707	1.227	0.679	4
T	1.001	0.087	0.880	0.275	-0.553	0.960	0.531	6
U	-0.468	-0.041	-0.412	-0.129	0.258	-0.449	-0.248	14
V	-0.496	-0.043	-0.436	-0.136	0.274	-0.476	-0.263	17

F represents the comprehensive score calculated by the variance-weighted method of the first six principal components.

## Discussion

### Genetic diversity of agronomic traits and commercial quality

Yield and commercial appearance are the primary determinants of adzuki bean production efficiency and market acceptance. However, systematic characterization of phenotypic variation in yield components and seed quality traits among mainstream Chinese adzuki bean cultivars remains limited. In this study, all six agronomic traits exhibited significant differences among cultivars (*P* < 0.05), with coefficients of variation (CV) ranging from 11.76% to 90.90%. Notably, pods per plant showed an extremely high CV of 90.90%, which was substantially higher than the values (35.2–68.7%) reported in previous studies involving regional germplasm collections (Wang et al., 2018; Hu et al., 2022). One possible reason for this high variation is that the tested varieties were collected from a wide geographic range (29.5°N to 45.6°N) with diverse ecological backgrounds; however, their actual environmental adaptability needs to be verified by multi-location trials. Additionally, the high sensitivity of podding traits to field microenvironmental factors (e.g., light interception and nutrient distribution) further amplified inter-varietal differences, a phenomenon also observed in mung bean and other leguminous crops (Ge et al., 2025). Grain yield of the tested varieties ranged from 833.49 to 1540.44 kg ha^−1^, revealing two distinct high-yielding strategies: Jihong 9218 exhibited a “multi-pod type” yield pattern, achieving high productivity through the maximum number of pods per plant; while Jihong 13 represented a “large-seed type” pattern, relying on the highest 100-seed weight. These two complementary yield formation patterns provide reference for breeding. It is speculated that multi-pod varieties may have application potential in high-density planting scenarios, and large-seed varieties may have better adaptability to mechanized harvesting and high-end grain markets, but these assumptions require dedicated field trials for validation. It is noteworthy that Henong 3, despite having the highest pod number, showed only moderate yield due to its extremely low 100-seed weight, confirming that balanced coordination among yield components is essential for maximizing grain yield (Bi et al., 2024; Delfim et al., 2025). For seed mineral nutrition and appearance quality, phosphorus (P) content showed the highest CV (58.57%), which may be related to both the heterogeneity of soil P availability and differences in P uptake and accumulation among varieties. Among seed color parameters, yellowness (b*) exhibited the greatest variation (CV = 51.86%), which has been closely associated with flavonoid and anthocyanin accumulation in the seed coat (Moon et al., 2026). This finding is consistent with the significant variation in total flavonoid content observed in our study. Notably, Jihong 9218 displayed superior performance in L*, a*, b* and chroma, indicating its excellent commercial value for the whole-grain market under the tested conditions.

### Processing, nutritional and functional quality variation

Processing adaptability is the core determinant of adzuki bean industrial value, with bean paste yield being the most critical indicator for the red bean paste industry, which accounts for over 70% of adzuki bean processing in China. In this study, Baihong 2 achieved a bean paste yield of 0.70, which was significantly higher than the average level (0.52–0.63) of previously reported widely cultivated varieties (Shi et al., 2026a). This favorable performance indicates that Baihong 2 is a promising candidate cultivar for red bean paste processing, and its industrial-scale application potential remains to be further verified. For textural properties, springiness showed the highest CV (57.23%), which is primarily governed by differences in starch granule structure and protein matrix composition among varieties. Baihong 2 also exhibited the lowest hardness, gumminess and chewiness, which was strongly correlated with its high amylose content and amylose/amylopectin (AM/AP) ratio. High amylose content promotes the formation of a loose and porous texture after cooking, which is an important factor affecting bean paste yield (Cao et al., 2012). The highest AM/AP ratio in Baihong 2 provides a potential mechanistic explanation for its favorable processing performance observed in this study. Among nutritional components, soluble sugar content showed an extremely high CV of 122.01%, indicating that this trait is strongly influenced by genotype-environment interactions and has substantial breeding potential. The AM/AP ratio varied from 0.11 to 0.29 (CV = 35.41%), which directly affects starch gelatinization properties and processing adaptability. This wide range provides valuable materials for breeding varieties tailored to different processing requirements, such as low-amylose varieties for canned products and high-amylose varieties for bean paste. For functional components, isoflavone monomers showed high phenotypic variation, with CV values of glycitin (77.84%), genistin (70.61%) and free daidzein (69.00%) all exceeding 65%. Combined with previous reports, it is speculated that isoflavone biosynthesis in legumes is mainly regulated by genetic factors, which needs to be further confirmed under multiple environmental conditions (Ji et al., 2026). Baohong 8824 showed outstanding performance in all isoflavone fractions, with total isoflavone content 32% higher than the average level of all tested varieties. Given the well-documented antioxidant, anti-inflammatory and estrogen-like activities of isoflavones (Zhang et al., 2026), this variety has promising application potential in functional food research and development, pending further validation of its bioavailability and functional efficacy. Kenjianhong had the highest total phenolic content, total flavonoid content and comprehensive antioxidant activity, consistent with the established view that phenolic compounds are the main contributors to the antioxidant capacity of legumes (Huang et al., 2025).

### Comprehensive quality evaluation and special-purpose variety screening

Principal component analysis (PCA) is a powerful multivariate statistical method that can effectively reduce data dimensionality and objectively evaluate comprehensive cultivar performance. In this study, the first six principal components explained 79.14% of the total variance, which is comparable to the cumulative variance contribution rates (72.3–81.5%) reported in previous crop quality evaluation studies (Chen et al., 2024; Shi et al., 2026b). Unlike most previous studies that only focused on 5–10 agronomic or nutritional traits, our PCA was based on 18 core indicators covering agronomic performance, mineral nutrition, processing quality, nutritional components and functional activities. This multidimensional quality evaluation system supports a more comprehensive phenotypic comparison of cultivars under the tested conditions, and provides reference for preliminary screening of cultivars for different applications. The comprehensive evaluation results identified Baihong 2, Jihong 8 and Jihong 13 as the top three varieties with the best overallperformance. Baihong 2 ranked first with a comprehensive score of 1.342, and its core advantage lies in its unparalleled processing quality, making it a promising candidate for red bean paste processing applications under the tested conditions. Jihong 8 and Jihong 13 showed balanced performance in yield, and quality traits, showing good comprehensive performance and application prospects for diversified utilization, which require further multi-environment verification. Importantly, our study also identified several special-purpose varieties with outstanding specific traits, even though they did not rank high in the comprehensive evaluation: Baohong 8824: Rich in isoflavones and GABA, with potential for functional food research and development; Kenjianhong: Superior antioxidant activity, with application potential for natural antioxidant extraction research; Jihong 9218: Highest yield and excellent commercial appearance, with good market application potential for general edible grain under the tested conditions. This classified preliminary screening strategy can provide reference for the targeted utilization of different varieties, and is expected to support the specialized and diversified development of the adzuki bean industry after further verification.

### Limitations and future research directions

This study provides the most comprehensive multi-dimensional quality evaluation of widely cultivated adzuki bean varieties in China to date and identified several promising candidate cultivars for different utilization directions. However, several limitations should be acknowledged: First, this study was conducted at a single location over a single growing season. Crop traits are significantly affected by environmental factors and genotype-environment (G×E) interactions. Therefore, the results only reflect the phenotypic performance of the tested varieties under Baoding 2025 conditions, and cannot support conclusions regarding nationwide superiority or G×E patterns. Multi-location and multi-year trials across major adzuki bean producing regions in China are needed to verify the stability of the identified favorable traits and evaluate the adaptability of these varieties to different ecological conditions. Second, this study did not measure anti-nutritional factors such as phytic acid and trypsin inhibitors, which can affect the nutritional value and digestibility of adzuki bean products. Future studies should supplement these indicators to obtain a more complete quality profile. Additionally, cooking quality traits such as cooking time and water absorption rate, which are important for household consumption, should also be included in the evaluation system. Third, this study only analyzed phenotypic correlations among traits, and the underlying molecular regulatory mechanisms remain unclear. Subsequent studies can integrate transcriptomics, metabolomics and genome-wide association studies (GWAS) to identify key genes and metabolic pathways controlling important quality traits such as bean paste yield and isoflavone accumulation. This will provide a theoretical basis for molecular marker-assisted breeding of adzuki bean.

## Conclusion

Using 22 widely cultivated adzuki bean varieties in China, this study performed a multidimensional quality evaluation under Baoding 2025 field conditions, and assessed their comprehensive performance via principal component analysis with 18 core indicators. Abundant phenotypic variation was observed across all traits, with pods per plant, isoflavone monomers and soluble sugar showing the highest variation. Baihong 2, Jihong 8 and Jihong 13 showed favorable comprehensive performance under the tested conditions, and three types of trait-specific candidate cultivars were preliminarily screened: high-yield Jihong 9218, processing-suitable Baihong 2, and functional-component-rich Baohong 8824 and Kenjianhong. This study established a preliminary quality evaluation framework, providing reference for cultivar screening, targeted breeding and industrial utilization; the environmental stability and application value of selected varieties require further multi-location and multi-year validation.

## Data Availability

The raw data supporting the conclusions of this article will be made available by the authors, without undue reservation.
